# The causal relationship of human blood metabolites with the components of Sarcopenia: a two-sample Mendelian randomization analysis

**DOI:** 10.1186/s12877-024-04938-x

**Published:** 2024-04-15

**Authors:** Wenxi Peng, Zhilin Xia, Yaxuan Guo, Linghong Li, Jianrong He, Yi Su

**Affiliations:** 1 Key Laboratory of Molecular Epidemiology of Hunan Province, School of Medicine, Hunan Normal University, 371 Tongzipo Road, Yuelu District, 410013 Changsha, Hunan China; 2 Division of Birth Cohort Study, Guangzhou Women and Children’s Medical Center, Guangzhou Medical University, 511436 Guangzhou, Guangdong China

**Keywords:** Hand grip strength, Walking pace, Appendicular lean mass, Metabolomics

## Abstract

**Background:**

Sarcopenia is a progressive loss of muscle mass and function. Since skeletal muscle plays a critical role in metabolic homeostasis, identifying the relationship of blood metabolites with sarcopenia components would help understand the etiology of sarcopenia.

**Methods:**

A two-sample Mendelian randomization study was conducted to examine the causal relationship of blood metabolites with the components of sarcopenia. Summary genetic association data for 309 known metabolites were obtained from the Twins UK cohort and KORA F4 study (7824 participants). The summary statistics for sarcopenia components [hand grip strength (HGS), walking pace (WP), and appendicular lean mass (ALM)] were obtained from the IEU Open GWAS project (461,089 participants). The inverse variance weighted method was used, and the MR-Egger, weighted median, and MR-PRESSO were used for the sensitivity analyses. Metabolic pathways analysis was further performed.

**Results:**

Fifty-four metabolites associated with sarcopenia components were selected from 275 known metabolites pool. Metabolites that are causally linked to the sarcopenia components were mainly enriched in amino sugar and nucleotide sugar metabolism, galactose metabolism, fructose and mannose metabolism, carnitine synthesis, and biotin metabolism. The associations of pentadecanoate (15:0) with ALM, and 3-dehydrocarnitine and isovalerylcarnitine with HGS were significant after Bonferroni correction with a threshold of *P* < 1.82 × 10^− 4^ (0.05/275). Meanwhile, the association of hyodeoxycholate and glycine with the right HGS, and androsterone sulfate with ALM were significant in the sensitivity analyses.

**Conclusion:**

Blood metabolites from different metabolism pathways were causally related to the components of sarcopenia. These findings might benefit the understanding of the biological mechanisms of sarcopenia and targeted drugs development for muscle health.

**Supplementary Information:**

The online version contains supplementary material available at 10.1186/s12877-024-04938-x.

## Introduction

Sarcopenia, an accelerated loss of muscle mass and function, is a progressive and complex disease associated with a higher risk of falls, frailty, morbidity, and mortality [1], which leads to decreased quality of life and a higher burden of healthcare [2]. Although sarcopenia has been recognized as a disease by the International Classification of Diseases (ICD) since 2016 [3], its biological mechanisms have not been fully understood.

Metabolites are intermediates or end products of metabolism that play essential roles in human health. Modern omics techniques, including metabolomics, have made positive contributions to exploring disease mechanisms, specifically providing novel insights into the biological mechanisms of diseases by revealing intermediate metabolites and altered metabolic pathways [4]. Recently, metabolomics has helped characterize specific metabolic phenotypes related to muscle health and explore the relationships between specific metabolites and muscle health. Metabolites biomarkers of L-alanine, gluconic acid, proline, and tryptophan were identified for severe sarcopenia in the community-dwelling older men [5], and pentadecanoic acid, 5’-Methylthioadenosine, asymmetric dimethylarginine, and glutamine were identified in diabetic patients with sarcopenia [6]. However, blood metabolites are probably susceptible to the confounding factors of diet, exercise and other lifestyle habits, which could not be clarified in traditional observational studies [7–9]. The causality of blood metabolites in sarcopenia needs valid confirmation.

Mendelian randomization (MR) analysis, using genetic variation as a natural experiment, is a useful strategy to investigate the causal relations between potentially modifiable exposures and health outcomes in observational studies [7]. Furthermore, two-sample MR could help estimate a causal effect of the risk factor on the outcome by incorporating different studies from multiple sources [8]. Then, based on the generated genetic and metabolic profiles, the causal associations of genetically determined metabolomics effects on muscle health could be explored.

Thus, this study aimed to investigate the potential causal relationships between the blood metabolites and the components of sarcopenia [hand grip strength (HGS), walking pace (WP), and appendicular lean mass (ALM)] using a two-sample MR approach. We further identified the potential metabolic pathways based on the metabolites with causal effects on sarcopenia components. Our study may help to understand the biological mechanisms of sarcopenia development.

## Materials and methods

### Study design

**Fig. 1:**
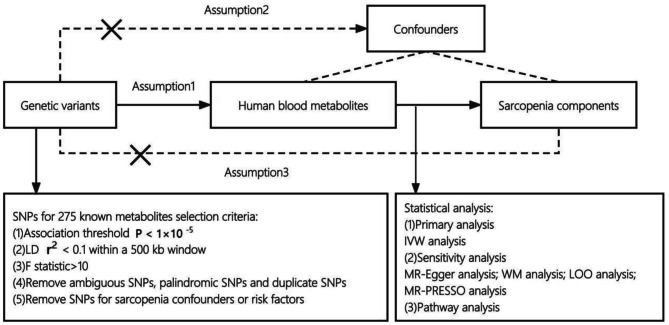
Overview of the current Mendelian randomization (MR) study. Notes: SNP, Single nucleotide polymorphism; LD, Linkage disequilibrium; IVW, Inverse-variance weighted; WM, Weighted median; LOO analysis, Leave-one-out sensitivity analysis; MR-PRESSO, Mendelian Randomization Pleiotropy RESidual Sum and Outlier. *Three assumptions that a Mendelian randomization study should satisfy: assumption 1, the genotype was related to the exposure(relevance assumption); assumption 2, the association of the genotype with the outcome was independent of the other confounding factors (independence assumption); assumption 3, the genotype was associated with the outcome only by the exposure studied (exclusivity assumption)

A two-sample MR design was applied, and the study methods complied with the STROBE-MR checklist [9]. Three assumptions that a Mendelian randomization study should satisfy: assumption 1, the genotype was related to the exposure (relevance assumption); assumption 2, the association of the genotype with the outcome was independent of the other confounding factors (independence assumption); assumption 3, the genotype was associated with the outcome only by the exposure studied (exclusivity assumption). An overview of the study design was shown in the Fig. 1.

### Data sources of exposure

Genome-wide association study (GWAS) data for blood metabolites were obtained from two European population cohorts [10], which included 1768 participants from the KORA F4 study in Germany and 6056 from the UK Twin Study. Table S1 in the Supplementary file 1 summarized the GWAS data used in this study. The database included a total of 529 metabolites profiled using liquid-phase chromatography and gas chromatography separation coupled with tandem mass spectrometry in either plasma or serum [10], which were chemically identified and could be assigned to eight broad metabolic groups (amino acids, carbohydrates, cofactors and vitamins, energy, lipid, nucleotides, peptides, and xenobiotics) [11]. After stringent quality controls, a subset of 452 metabolites were available for genetic analysis, including 275 known metabolites.

### Selection of instrumental variables (IVs) for blood metabolites

The IVs for each of the 275 known metabolites were constructed separately. Several procedures were performed to ensure the assumption that the genotype was related to the exposure: (a) the genetic variants were identified with the association at a threshold of *P* < 1 × 10 ^− 5^ in the MR Analysis. (b) independent variants were identified using a clumping procedure implemented in R software, in which a linkage-disequilibrium (LD) threshold of r^2^ < 0.1 within a 500 kb window in the European 1000 Genomes Project Phase 3 reference panel was set. Single nucleotide polymorphisms (SNPs) absent from the LD reference panel were also removed. Instrument SNPs were selected by removing SNPs with minor allele frequency (MAF) less than 0.01. Ambiguous SNPs (e.g., A/G vs. A/C) and palindromic SNPs (i.e., A/T or G/C) were directly excluded during the harmonizing process to ensure that the effect of each SNP on the exposure and its effect on the outcome corresponds to the same allele. Next, for the association of each SNP with each metabolite, the F statistic and R square were calculated, respectively. The amount of variance explained by the IVs was calculated for each exposure using the TwoSampleMR package (get_r_from_bsen function). The potential weak instrumental variable bias was tested by calculating the F statistic using the formula F = beta^2^ / se^2^, where beta is the estimated genetic effect on human blood metabolites, and se is the standard error of the genetic effect [12]. The possibility of weak IV bias was slight when the F statistic was much greater than 10 [13]. (c) Lastly, potential pleiotropic effects of the SNPs used as IVs were tested using the online tools LDtrait [14] and PhenoScanner [15]. The SNPs that were significantly associated with the confounders or risk factor traits of sarcopenia, such as BMI, obesity, diabetes, chronic inflammatory disease, older age, low socioeconomic status, poor diet, low physical activity, and lack of physical activity, were removed. The stringently selected SNPs above were used as the IVs in the two-sample MR analysis subsequently.

### Data sources of outcome

The data of sarcopenia components were obtained from published studies from the UK Biobank [16]. The GWAS-associated data for HGS included 461,089 individuals for the right HGS and 461,026 individuals for the left HGS [17]. Genetic predictors of WP were assessed using the summary statistics from the UK Biobank, which includes 459,915 individuals of European ancestry [17]. The categorical variable was further defined according to WP (slow pace: WP < 3 mph, moderate pace: 3 ≤ WP ≤ 4 mph, and fast pace: WP>4 mph). The GWAS-associated data for ALM included 450,243 individuals from the European Bioinformatics Institute (EBI) database [18].

### Statistical analysis

#### MR analysis

The causal associations between the 275 known human blood metabolites and the components of sarcopenia were systematically assessed by a two-sample MR analysis. The inverse variance weighting (IVW) method was used to evaluate the causal effects in the two-sample MR analysis. A fixed effect model was used if there was no heterogeneity and no pleiotropy, and a random effect model was used if there was a heterogeneity but no pleiotropy. The Cochran Q test was carried out to detect the existence of heterogeneity, with the Cochran-Q derived *P* < 0.05 and I^2^ > 25% recognized as a heterogeneity [19]. The estimates of IVW were obtained by calculating the slope of the weighted linear regression [20]. A multiple-testing-adjusted threshold using the Bonferroni correction was adopted to declare a statistically significant causal relationship. The associated metabolites identified at a threshold of *P* < 0.05 but did not reach the Bonferroni-corrected significance, were also suggested as potential risk factors for the components of sarcopenia.

#### Sensitivity analyses

Sensitivity analyses were performed to assess any bias in the MR assumptions. The MR-Egger method was used to test the directional horizontal pleiotropy and to estimate the causal effects if there were pleiotropies or any violations of the IVs assumptions [21]. Weighted median estimates remain valid even when up to 50% of the information was derived from the valid SNPs [22]. A leave-one-out sensitivity analysis was further performed to determine whether the estimates were influenced by a single SNP [21]. MR-PRESSO was used to examine the horizontal pleiotropy outliers and to provide corrected estimates [23]. Additionally, the MR Steiger directionality test was performed to see whether the results supported the proposed hypothesis. *P* < 0.05 was considered statistically significant. All MR analyses were conducted using R software (R Core Team 2022, version 4.2.1) with the R package “TwoSample MR package” (version 0.5.6) and “MR-PRESSO” (version 1.0).

#### Metabolic pathway analysis

A metabolic pathway analysis for the identified metabolites was performed using the web-based MetaboAnalyst 5.0 software [24]. https://www.metaboanalyst.ca/docs/Publications.xhtml. The functional enrichment analysis module and pathway analysis module were used to perform metabolic pathway analysis for the blood metabolites that were identified by the IVW method, as mentioned above. The metabolic pathway analysis tested 183 human metabolic pathways from two metabolite set libraries, including 99 metabolite sets from the Small Molecule Pathway Database (SMPDB) and 84 metabolite sets from the Kyoto encyclopedia of genes and genomes (KEGG) [4].

## Results

This study screened 5891 SNPs linked to 275 metabolites which were included in the MR analysis. There were 17 SNPs that were significantly associated with the aforementioned confounders or risk factors, including BMI, obesity, diabetes, chronic inflammatory disease, were removed (Supplementary file 1: Table S2 and Table S3). Then, 1015 IVs having a potential causal relationship with the components of sarcopenia were selected from the GWAS datasets of 275 metabolites, and then available for further MR analysis (Supplementary file 1: Table S2 and S4). These IVs could explain 0.25–10.02% of the variance of their corresponding metabolites. These IVs had a minimum F-statistic of 18.63, indicating that all the IVs were valid in the MR Analysis (F statistics > 10).

Fifty-four genetically predicted known metabolites associated with the components of sarcopenia were observed at the significance of *P* < 0.05 in the IVW analysis (Supplementary file 2: Figure S1). As indicated by the results from the MR Steiger directionality test, the current estimates of causal direction were accurate (*P* < 0.001), and no SNP had shown pleiotropy (Supplementary file 1: Table S5). Among these, 19 known metabolites were associated with two or more components of sarcopenia simultaneously when *P* < 0.05 was used as the threshold (Supplementary file 1: Table S6).

**Fig. 2:**
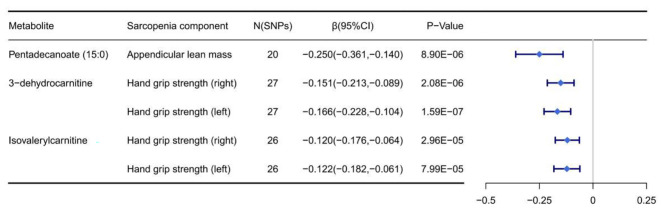
The significant Mendelian randomization (MR) association (*P* < 1.82 × 10^− 4^) between known metabolites and components of sarcopenia

After the multiple-testing-adjusted Bonferroni correction with a threshold of 1.82 × 10^− 4^ (0.05/275), 5 causal associations between 3 metabolites and sarcopenia components were observed. The increased Pentadecanoate (15:0) [β(95%) = -0.250 (-0.361, -0.140), *P* = 8.90 × 10^− 6^] was associated with a decrease in ALM. 3-dehydrocarnitine [β(95%) = -0.151(-0.213, -0.089), *P* = 2.08 × 10^− 6^] and isovalerylcarnitine [β(95%) = -0.120(-0.176, -0.064), P = 2.96 × 10^− 5^] were negatively associated with right HGS, while 3-dehydrocarnitine [β(95%) =-0.166(-0.228, -0.104), P = 1.59 × 10^− 7^] and isovalerylcarnitine [β(95%) = -0.122(-0.182, -0.061), *P* = 7.99 × 10^− 5^] were negatively associated with left HGS (Fig. 2 and Supplementary file 1: Table S7).

### Sensitivity analyses

**Fig. 3:**
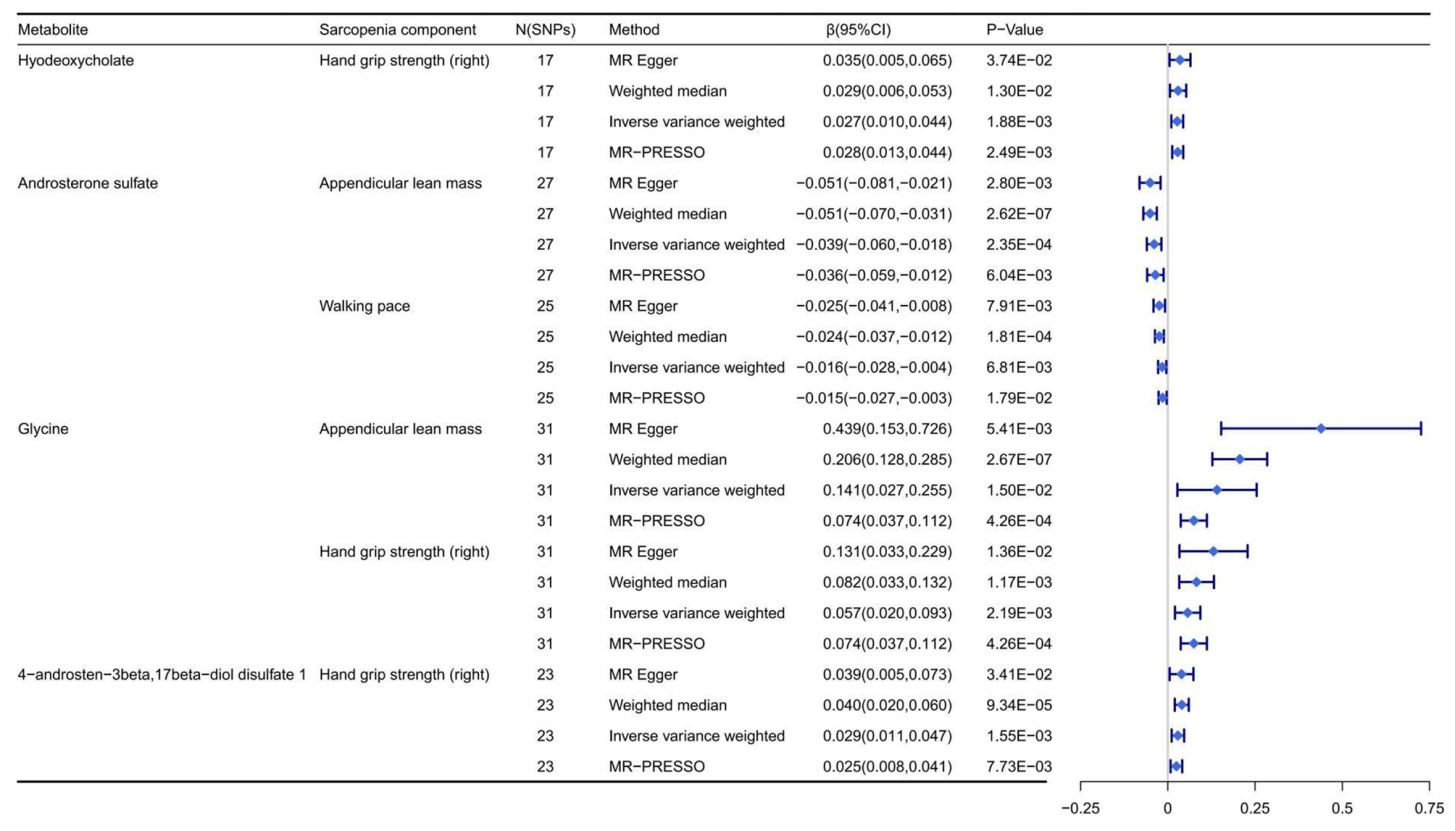
The known metabolites validated in the sensitivity analyses

Sensitivity analyses were conducted for the identified metabolites to evaluate the robustness of the estimates. The causal relationships of androsterone sulfate and glycine with ALM, hyodeoxycholate, glycine and 4-androsten-3beta, 17beta-diol disulfate 1 with the right HGS, and androsterone sulfate with WP were reliable and similar effect estimates were found for the weighted median, MR-Egger and MR-PRESSO method (Fig. 3 and Supplementary file 1: Table S8). According to the results of the leave-one-out sensitivity analysis, hyodeoxycholate [β(95%) = 0.027(0.010, 0.044), *P* = 1.88 × 10^− 3^] and glycine [β(95%) = 0.057 (0.020,0.093), P = 2.19 × 10^− 3^] increased was associated with an increase estimate of right HGS, and androsterone sulfate showed a significant negative associated with ALM[β(95%) = -0.039(-0.060, -0.018), P = 2.35 × 10^− 4^]. Therefore, the MR analysis was reliable, and no single SNPs changed the results substantially (Supplementary file 2: Figure S2-S5).

### Metabolic pathway analysis

Thirteen metabolic pathways associated with sarcopenia components were identified by the metabolic pathway analysis (Table 1). Pathways enriched for metabolites associated were amino sugar and nucleotide sugar metabolism, galactose metabolism, fructose and mannose metabolism, carnitine synthesis, biotin metabolism. The pathways identified in the functional enrichment analysis module are shown in Supplementary file 1: Table S9.

**Table 1:** The metabolic pathways identified in relation to the components of sarcopenia

Sarcopenia component	Metabolic pathways	Involved metabolites	*P*-Value	Details
Hand grip strength (right)	Beta Oxidation of Very Long Chain Fatty Acids	L-Acetylcarnitine	3.82E-02	SMPDB
	Alanine Metabolism	Glycine	4.11E-02	SMPDB
	Carnitine Synthesis	Glycine	4.69E-02	SMPDB
Walking pace	Alpha Linolenic Acid and Linoleic Acid Metabolism	Eicosapentaenoic acid; Docosapentaenoic acid (22n-6)	3.41E-03	SMPDB
	Fructose and Mannose Degradation	D-Fructose; D-Mannose	1.04E-02	SMPDB
	Galactose Metabolism	D-Fructose; D-Mannose	1.27E-02	SMPDB
	Fructose and mannose metabolism	D-Fructose; D-Mannose	4.56E-04	KEGG
	Galactose metabolism	D-Fructose; D-Mannose	8.41E-04	KEGG
	Amino sugar and nucleotide sugar metabolism	D-Fructose	2.05E-03	KEGG
	Starch and sucrose metabolism	D-Fructose; D-Mannose	3.39E-02	KEGG
Appendicular lean mass	Carnitine Synthesis	L-Lysine; Oxoglutaric acid	3.41E-03	SMPDB
	Lysine Degradation	L-Lysine; Oxoglutaric acid	5.34E-03	SMPDB
	Citric Acid Cycle	Citric acid; Oxoglutaric acid	8.99E-03	SMPDB
	Fructose and Mannose Degradation	D-Fructose; D-Mannose	1.04E-02	SMPDB
	Galactose Metabolism	D-Fructose; D-Mannose	1.27E-02	SMPDB
	Warburg Effect	Citric acid; Oxoglutaric acid	3.06E-02	SMPDB
	Biotin Metabolism	L-Lysine	4.10E-02	SMPDB
	Malate-Aspartate Shuttle	Oxoglutaric acid	4.10E-02	SMPDB
	Fructose and mannose metabolism	D-Fructose; D-Mannose	2.23E-03	KEGG
	Citrate cycle (TCA cycle)	2-Oxoglutarate; Citrate	2.23E-03	KEGG
	Galactose metabolism	D-Fructose; D-Mannose	4.07E-03	KEGG
	Alanine, aspartate and glutamate metabolism	2-Oxoglutarate; Citrate	4.38E-03	KEGG
	Amino sugar and nucleotide sugar metabolism	D-Fructose; D-Mannose	9.73E-03	KEGG
	Biotin metabolism	L-Lysine	3.76E-02	KEGG

Notes: KEGG, Kyoto encyclopedia of genes and genomes; SMPDB, Small molecule pathway database.

## Discussion

This study assessed the causal relationships between the blood metabolites and the sarcopenia-related traits through a MR study combining genomics and metabolomics. After the multiple-testing-adjusted Bonferroni correction, 3 known metabolites, which were pentadecanoate (15:0) on ALM, 3-dehydrocarnitine and isovalerylcarnitine on HGS were identified. Meanwhile, hyodeoxycholate and glycine were reliably positively associated with the right HGS, and androsterone sulfate showed a reliable negative association with ALM in the sensitivity analysis. 13 metabolic pathways were identified to be causally associated with the components of sarcopenia.

Pentadecanoate (15:0) was a dietary biomarkers for dairy-fat consumption [25], which also played a vital role in muscle metabolism and function [26]. This is consistent to the findings in the current study. Isobutyrylcarnitine is a metabolic product that occurs during the transfer of acyl residues from isobutyryl coenzyme A to carnitine [27]. Previous studies have shown that higher levels of hydroxylated acylcarnitine was negatively correlated with the decline in grip strength in a short term [34], while medium and long-chain acylcarnitines were correlated with a poorer physical function [28]. Acylcarnitine, disrupting peroxisomal or mitochondrial oxidation processes [29], also plays a part in mitochondrial function, whose dysregulation is highly involved in the pathological loss of skeletal muscle mass and function in the elderly [30]. 3-dehydrocarnitine is an intermediate in carnitine degradation, and carnitine has been linked to muscle mass or physical weakness [31, 32], which plays a crucial role in energy metabolism and mediates the pathway of fatty acid oxidation in the mitochondria [33]. However, these observational results are limited in determining causal relationships. Therefore, further investigations are warranted to confirm the biological functions of the above metabolites in relation to the muscle health.

Hyodeoxycholate, androsterone sulfate and glycine have also been identified as certain specific metabolites in relation to muscle health in the current study. Hyodeoxycholate is a bile acid derivative, and a negative correlation between blood hyodeoxycholic acid and muscle uncoupling protein 3 gene was found, suggesting a potential impact on muscle function [34]. Androsterone sulfate is one metabolite of androgens, which may also be associated with the change of lean body mass and muscle mass [35]. This study found a negative association between androsterone sulfate and ALM, which further supports the potential link between androsterone sulfate and muscle function. Glycine is involved in anti-inflammatory, immune function, and antioxidant responses. Although glycine was found to be negatively associated with HGS in older men in a cross-sectional study [28], the nutritional supplement of glycine reversed multiple age-related abnormalities. It promoted the health of older participants in a clinical trial [36]. In the current study, glycine was also found to be causally and positively related to the HGS, which could help understand the positive effect of glycine.

Metabolic pathway analysis revealed that amino sugar and nucleotide sugar metabolism, galactose metabolism, fructose and mannose metabolism, carnitine synthesis, biotin metabolism were critical pathways in relation to muscle health. Amino sugar and nucleotide sugar metabolism is involved in oxidative induction and inhibits muscle glucose uptake, which may have a potential regulatory effect on blood glucose levels [37]. A genetic study identified the critical genes involved in muscle growth modification development by bioinformatics analysis, and the differentially expressed genes were mainly involved in skeletal muscle contraction, fatty acid metabolism, and galactose metabolism [38]. Additionally, the effect of fructose and mannose metabolism pathway was identified in the current study, which may be related to its effect on the metabolome of myopathies. And fructose and mannose metabolism were found to be closely related to glycolysis and may provide substrates for sugar nucleotide synthesis in the previous studies [39], which may interact with the energy metabolism in muscle.

This study has several advantages. At first, a wide range of blood metabolites have been explored to investigate the potential metabolic profile causally correlated with the value of muscle health. Secondly, a two-sample MR Design was applied to exclude reverse causality and residual confounding, and the consistent results from various MR Models helped verify the MR assumptions and support the robustness of the MR estimates. Thirdly, the SNPs associated with potential confounders were evaluated and excluded. Finally, the potential metabolite groups or pathways were explored additionally to help understand the biological processes of muscle health. This study has several limitations. Firstly, due to limited resources, no causal relationship has been identified between blood metabolites and sarcopenia diagnosed based on the cut-off values [40, 41]. Because more phenotypic information cannot be used to study individuals, the results lacked the influence on body size and composition. Secondly, more IVs identified in GWAS might be needed to help accurately assess the genetic influence on metabolites. The third approximation of muscle mass in the UK Biobank used in the present study was measured using bio-impedance analysis (BIA), which may be less accurate than the values measured by other imaging detections, such as dual-energy x-ray absorptiometry (DXA), magnetic resonance imaging (MRI) and computed tomography (CT). In addition, demographic characteristics had not been considered in the present analyses, and the study was primarily limited to individuals of European ancestry, which limits the generalization of the findings.

## Conclusion

In conclusion, generally, the metabolites causally linked to the sarcopenia components were mainly enriched in the pathway of amino sugar and nucleotide sugar metabolism, galactose metabolism, fructose and mannose metabolism, carnitine synthesis, and biotin metabolism. Several metabolites were further identified by Bonferroni correction. Pentadecanoate (15:0) was negatively associated with the estimate of ALM. 3-dehydrocarnitine and isovalerylcarnitine were negatively associated with HGS. Meanwhile, the association of hyodeoxycholate and glycine with the right HGS, and androsterone sulfate with ALM were significant in the sensitivity analyses. These findings might have implications for the biological mechanisms of sarcopenia and targeted drug development for muscle health.

### Electronic supplementary material

Below is the link to the electronic supplementary material.

Supplementary Material 1

Supplementary Material 2

Supplementary Material 3

## Data Availability

Publicly available datasets were analyzed in this study. This data can be found here: (https://gwas.mrcieu.ac.uk/) and (http://app.mrbase.org/).

## Declarations

### AI and AI-assisted technologies in the writing process

The authors declare they did not use AI and AI-assisted technologies in the writing process.

## Abbreviations

MR: Mendelian randomization
KORA: Cooperative Health Research in the Region of Augsburg
HGS: Hand grip strength
WP: Walking pace
ALM: Appendicular lean mass
IEU: Integrative Epidemiology Unit
IVW: Inversevariance weighted
MR-PRESSO: Mendelian Randomization Pleiotropy RESidual Sum and Outlier
ICD: International Statistical Classification of Diseases
GWAS: Genome-wide association study
KEGG: Kyoto encyclopedia of genes and genomes
SMPDB: Small molecule pathway database
IVs: Instrumental variables
LD: Linkage disequilibrium
SNP: Single nucleotide polymorphism
LOO: Leave-one-out sensitivity analysis
BIA: Bio-impedance analysis
MRI: Magnetic resonance imaging
CT: Computed tomography
