# Supplementary material for: The causal relationship of human blood metabolites with the components of Sarcopenia: a two-sample Mendelian randomization analysis
Source: BMC Geriatr. 2024 Apr 15;24:339. doi: 10.1186/s12877-024-04938-x (PMC11017669; doi:10.1186/s12877-024-04938-x)
Supplement: Supplementary file 1 — Supplementary Material 1 [file 12877_2024_4938_MOESM1_ESM.docx]

**STROBE-MR checklist of recommended items to address in reports of Mendelian randomization studies**^1^ ^2^

| **Item No.** | **Section** | **Checklist item** | **Page No.** | **Relevant text from manuscript** |
| --- | --- | --- | --- | --- |
| 1 | **TITLE and ABSTRACT** | Indicate Mendelian randomization (MR) as the study’s design in the title and/or the abstract if that is a main purpose of the study | 1 | The causal relationship of human blood metabolites with the components of sarcopenia: a two-sample Mendelian randomization analysis |
|  | **INTRODUCTION** |  |  |  |
| 2 | **Background** | Explain the scientific background and rationale for the reported study. What is the exposure? Is a potential causal relationship between exposure and outcome plausible? Justify why MR is a helpful method to address the study question | 4-5 | Recently, metabolomics has helped characterize specific metabolic phenotypes related to muscle health and explore the relationships between specific metabolites and muscle health......  Mendelian randomization (MR) analysis, using genetic variation as a natural experiment, is a useful strategy to investigate the causal relations between potentially modifiable exposures and health outcomes in observational studies...... |
| 3 | **Objectives** | State specific objectives clearly, including pre-specified causal hypotheses (if any). State that MR is a method that, under specific assumptions, intends to estimate causal effects | 5 | Thus, this study aimed to investigate the potential causal relationships between the blood metabolites and the components of sarcopenia [hand grip strength (HGS), walking pace (WP), and appendicular lean mass (ALM)] using a two-sample MR approach. We further identified the potential metabolic pathways based on the metabolites with causal effects on sarcopenia components. Our study may help to understand the biological mechanisms of sarcopenia development. |
|  | **METHODS** |  |  |  |
| 4 | **Study design and data sources** | Present key elements of the study design early in the article. Consider including a table listing sources of data for all phases of the study. For each data source contributing to the analysis, describe the following: |  |  |
|  | a) | Setting: Describe the study design and the underlying population, if possible. Describe the setting, locations, and relevant dates, including periods of recruitment, exposure, follow-up, and data collection, when available. | 5-8 | Detailed information, such as recruitment criteria of population and quality control of genetic data, can be found in the original paper. |
|  | b) | Participants: Give the eligibility criteria, and the sources and methods of selection of participants. Report the sample size, and whether any power or sample size calculations were carried out prior to the main analysis | 5-8 | Detailed information, such as recruitment criteria of population and quality control of genetic data, can be found in the original paper. |
|  | c) | Describe measurement, quality control and selection of genetic variants | 5-8 | Detailed information, such as recruitment criteria of population and quality control of genetic data, can be found in the original paper. |
|  | d) | For each exposure, outcome, and other relevant variables, describe methods of assessment and diagnostic criteria for diseases | 5-8 | Genome-wide association study (GWAS) data for blood metabolites were obtained from two European population cohorts (10), which included 1768 participants from the KORA F4 study in Germany and 6056 from the UK Twin Study. Table S1 in the Supplementary file 1 summarized the GWAS data used in this study. The database included a total of 529 metabolites profiled using liquid-phase chromatography and gas chromatography separation coupled with tandem mass spectrometry in either plasma or serum (10), which were chemically identified and could be assigned to eight broad metabolic groups (amino acids, carbohydrates, cofactors and vitamins, energy, lipid, nucleotides, peptides, and xenobiotics) (11). After stringent quality controls, a subset of 452 metabolites waswere available for genetic analysis, including 275 known metabolites.  The data of sarcopenia components were obtained from published studies from the UK Biobank (16). The GWAS-associated data for HGS included 461,089 individuals for the right HGS and 461,026 individuals for the left HGS (17). Genetic predictors of WP were assessed using the summary statistics from the UK Biobank, which includes 459,915 individuals of European ancestry (17). The categorical variable was further defined according to WP (slow pace: WP < 3 mph, moderate pace: 3 ≤ WP ≤ 4 mph, and fast pace: WP＞4 mph). The GWAS-associated data for ALM included 450,243 individuals from the European Bioinformatics Institute (EBI) database (18). |
|  | e) | Provide details of ethics committee approval and participant informed consent, if relevant | No Applicable | These cohort studies were approved by the local ethics committees, and all participants provided their informed consents. Thus, no additional ethics approval was provided in the present study. |
| 5 | **Assumptions** | Explicitly state the three core IV assumptions for the main analysis (relevance, independence and exclusion restriction) as well assumptions for any additional or sensitivity analysis | 5 | Three assumptions that a Mendelian randomization study should satisfy: assumption 1, the genotype was related to the exposure (relevance assumption); assumption 2, the association of the genotype with the outcome was independent of the other confounding factors (independence assumption); assumption 3, the genotype was associated with the outcome only by the exposure studied (exclusivity assumption). |
| 6 | **Statistical methods: main analysis** | Describe statistical methods and statistics used |  |  |
|  | a) | Describe how quantitative variables were handled in the analyses (i.e., scale, units, model) |  | Supplementary file 1: Table S1 |
|  | b) | Describe how genetic variants were handled in the analyses and, if applicable, how their weights were selected | 6-7 | The IVs for each of the 275 known metabolites were constructed separately. Several procedures were performed to ensure the assumption that the genotype was related to the exposure: (a) the genetic variants were identified with the association at a threshold of P < 1×10 -5 in the MR Analysis. (b) independent variants were identified using a clumping procedure implemented in R software, in which a linkage-disequilibrium (LD) threshold of r2  <  0.1 within a 500 kb window in the European 1000 Genomes Project Phase 3 reference panel was set. Single nucleotide polymorphisms (SNPs) absent from the LD reference panel were also removed. Instrument SNPs were selected by removing SNPs with minor allele frequency (MAF) less than 0.01. Ambiguous SNPs (e.g., A/G vs. A/C) and palindromic SNPs (i.e., A/T or G/C) were directly excluded during the harmonizing process to ensure that the effect of each SNP on the exposure and its effect on the outcome corresponds to the same allele. Next, for the association of each SNP with each metabolite, the F statistic and R square were calculated, respectively. The amount of variance explained by the IVs was calculated for each exposure using the TwoSampleMR package (get_r_from_bsen function). The potential weak instrumental variable bias was tested by calculating the F statistic using the formula F = beta2 / se2, where beta is the estimated genetic effect on human blood metabolites, and se is the standard error of the genetic effect (12). The possibility of weak IV bias was slight when the F statistic was much greater than 10 (13). (c) Lastly, potential pleiotropic effects of the SNPs used as IVs were tested using the online tools LDtrait (14) and PhenoScanner (15). The SNPs that were significantly associated with the confounders or risk factor traits of sarcopenia, such as BMI, obesity, diabetes, chronic inflammatory disease, older age, low socioeconomic status, poor diet, low physical activity, and lack of physical activity etc., were removed. The stringently selected SNPs above were used as the IVs in the two-sample MR analysis subsequently. |
|  | c) | Describe the MR estimator (e.g. two-stage least squares, Wald ratio) and related statistics. Detail the included covariates and, in case of two-sample MR, whether the same covariate set was used for adjustment in the two samples | 5-8 | The list of covariates varies between original GWASs, but always included sex and age. Details can be found in the original studies. |
|  | d) | Explain how missing data were addressed | 5-8 | Detailed information, such as recruitment criteria of population and quality control of genetic data, can be found in the original paper. |
|  | e) | If applicable, indicate how multiple testing was addressed | 8 | Bonferroni correction is a threshold of P < 1.82 × 10^−4^ (0.05/275), but not reach the Bonferroni-corrected significance, were also suggested as potential risk factors for the components of sarcopenia. |
| 7 | **Assessment of assumptions** | Describe any methods or prior knowledge used to assess the assumptions or justify their validity | 10-11 | The inverse variance weighting (IVW) method was used to evaluate the causal effects in the two-sample MR analysis. A fixed effect model was used if there was no heterogeneity and no pleiotropy, and a random effect model was used if there was a heterogeneity but no pleiotropy. The Cochran Q test was carried out to detect the existence of heterogeneity, with the Cochran-Q derived P < 0.05 and I^2^ > 25% recognized as a heterogeneity (19). The estimates of IVW were obtained by calculating the slope of the weighted linear regression (20). A multiple-testing-adjusted threshold using the Bonferroni correction was adopted to declare a statistically significant causal relationship. The associated metabolites identified at a threshold of P < 0.05 but did not reach the Bonferroni-corrected significance, were also suggested as potential risk factors for the components of sarcopenia. |
| 8 | **Sensitivity analyses and additional analyses** | Describe any sensitivity analyses or additional analyses performed (e.g. comparison of effect estimates from different approaches, independent replication, bias analytic techniques, validation of instruments, simulations) | 11-12 | Sensitivity analyses were performed to assess any bias in the MR assumptions. The MR-Egger method was used to test the directional horizontal pleiotropy and to estimate the causal effects if there were pleiotropies or any violations of the IVs assumptions (21). Weighted median estimates remain valid even when up to 50% of the information was derived from the valid SNPs (22). A leave-one-out sensitivity analysis was further performed to determine whether the estimates were influenced by a single SNP (21). MR-PRESSO was used to examine the horizontal pleiotropy outliers and to provide corrected estimates (23). Additionally, the MR Steiger directionality test was performed to see whether the results supported the proposed hypothesis. |
| 9 | **Software and pre-registration** |  |  |  |
|  | a) | Name statistical software and package(s), including version and settings used | 9 | All MR analyses were conducted using R software (R Core Team 2022, version 4.2.1) with the R package “TwoSample MR package” (version 0.5.6) and “MR-PRESSO” (version 1.0). |
|  | b) | State whether the study protocol and details were pre-registered (as well as when and where) | No Applicable | This is a secondary analysis based on summary statistics from existing, published studies. The ethical approval and informed consent have been obtained by all original studies. |
|  | **RESULTS** |  |  |  |
| 10 | **Descriptive data** |  |  |  |
|  | a) | Report the numbers of individuals at each stage of included studies and reasons for exclusion. Consider use of a flow diagram | 5-8 | Detailed information, such as recruitment criteria of population and quality control of genetic data, can be found in the original paper . |
|  | b) | Report summary statistics for phenotypic exposure(s), outcome(s), and other relevant variables (e.g. means, SDs, proportions) | 5-8 | Details can be found in the original studies. |
|  | c) | If the data sources include meta-analyses of previous studies, provide the assessments of heterogeneity across these studies | 5-8 | Detailed information, such as recruitment criteria of population and quality control of genetic data, can be found in the original paper. |
|  | d) | For two-sample MR:  i.  Provide justification of the similarity of the genetic variant-exposure associations between the exposure and outcome samples  ii.  Provide information on the number of individuals who overlap between the exposure and outcome studies | 5-8 | These GWAS sample populations needed to be predominantly of European descent and largely independent of each other. |
| 11 | **Main results** |  |  |  |
|  | a) | Report the associations between genetic variant and exposure, and between genetic variant and outcome, preferably on an interpretable scale | 10-11 | MR-PRESSO Mendelian Randomization Pleiotropy RESidual Sum and Outlier |
|  | b) | Report MR estimates of the relationship between exposure and outcome, and the measures of uncertainty from the MR analysis, on an interpretable scale, such as odds ratio or relative risk per SD difference | 10-11 | Fifty-four genetically predicted known metabolites associated with the components of sarcopenia were observed at the significance of P < 0.05 in the IVW analysis (Supplementary file 2: Figure S1). As indicated by the results from the MR Steiger directionality test, the current estimates of causal direction were accurate (P < 0.001), and no SNP had shown pleiotropy (Supplementary file 1: Table S5). Among these, 19 known metabolites were associated with two or more components of sarcopenia simultaneously when P < 0.05 was used as the threshold (Supplementary file 1: Table S6).  After the multiple-testing-adjusted Bonferroni correction with a threshold of 1.82 × 10^−4^ (0.05/275), 5 causal associations between 3 metabolites and sarcopenia components were observed. The increased Pentadecanoate (15:0) [β(95%) = -0.250 (-0.361, -0.140), P = 8.90×10^-6^] was associated with a decrease in ALM. 3-dehydrocarnitine [β(95%) = -0.151(-0.213, -0.089), P = 2.08×10^-6^] and isovalerylcarnitine [β(95%) =-0.166(-0.228, -0.104), P = 1.59×10-7] were negatively associated with right HGS, while 3-dehydrocarnitine [β(95%) = -0.120(-0.176, -0.064), P = 2.96×10^-5^] and isovalerylcarnitine [β(95%) = -0.122(-0.182, -0.061), P = 7.99×10^-5^] were negatively associated with left HGS (Figure 2 and Supplementary file 1: Table S7). |
|  | c) | If relevant, consider translating estimates of relative risk into absolute risk for a meaningful time period | No Applicable |  |
|  | d) | Consider plots to visualize results (e.g. forest plot, scatterplot of associations between genetic variants and outcome versus between genetic variants and exposure) |  | Figure 2-3, Supplementary file 1: Figure S1-5. |
| 12 | **Assessment of assumptions** |  |  |  |
|  | a) | Report the assessment of the validity of the assumptions | 10-11 | Fifty-four genetically predicted known metabolites associated with the components of sarcopenia were observed at the significance of P < 0.05 in the IVW analysis (Supplementary file 2: Figure S1). As indicated by the results from the MR Steiger directionality test, the current estimates of causal direction were accurate (P < 0.001), and no SNP had shown pleiotropy (Supplementary file 1: Table S5). Among these, 19 known metabolites were associated with two or more components of sarcopenia simultaneously when P < 0.05 was used as the threshold (Supplementary file 1: Table S6).  After the multiple-testing-adjusted Bonferroni correction with a threshold of 1.82 × 10^−4^ (0.05/275), 5 causal associations between 3 metabolites and sarcopenia components were observed. The increased Pentadecanoate (15:0) [β(95%) = -0.250 (-0.361, -0.140), P = 8.90×10^-6^] was associated with a decrease in ALM. 3-dehydrocarnitine [β(95%) = -0.151(-0.213, -0.089), P = 2.08×10^-6^] and isovalerylcarnitine [β(95%) =-0.166(-0.228, -0.104), P = 1.59×10-7] were negatively associated with right HGS, while 3-dehydrocarnitine [β(95%) = -0.120(-0.176, -0.064), P = 2.96×10^-5^] and isovalerylcarnitine [β(95%) = -0.122(-0.182, -0.061), P = 7.99×10^-5^] were negatively associated with left HGS (Figure 2 and Supplementary file 1: Table S7). |
|  | b) | Report any additional statistics (e.g., assessments of heterogeneity across genetic variants, such as *I^2^*, Q statistic or E-value) | 10-11 | Fifty-four genetically predicted known metabolites associated with the components of sarcopenia were observed at the significance of P < 0.05 in the IVW analysis (Supplementary file 2: Figure S1). As indicated by the results from the MR Steiger directionality test, the current estimates of causal direction were accurate (P < 0.001), and no SNP had shown pleiotropy (Supplementary file 1: Table S5). Among these, 19 known metabolites were associated with two or more components of sarcopenia simultaneously when P < 0.05 was used as the threshold (Supplementary file 1: Table S6).  After the multiple-testing-adjusted Bonferroni correction with a threshold of 1.82 × 10^−4^ (0.05/275), 5 causal associations between 3 metabolites and sarcopenia components were observed. The increased Pentadecanoate (15:0) [β(95%) = -0.250 (-0.361, -0.140), P = 8.90×10^-6^] was associated with a decrease in ALM. 3-dehydrocarnitine [β(95%) = -0.151(-0.213, -0.089), P = 2.08×10^-6^] and isovalerylcarnitine [β(95%) =-0.166(-0.228, -0.104), P = 1.59×10-7] were negatively associated with right HGS, while 3-dehydrocarnitine [β(95%) = -0.120(-0.176, -0.064), P = 2.96×10^-5^] and isovalerylcarnitine [β(95%) = -0.122(-0.182, -0.061), P = 7.99×10^-5^] were negatively associated with left HGS (Figure 2 and Supplementary file 1: Table S7). |
| 13 | **Sensitivity analyses and additional analyses** |  |  |  |
|  | a) | Report any sensitivity analyses to assess the robustness of the main results to violations of the assumptions | 11-12 | Sensitivity analyses were conducted for the identified metabolites to evaluate the robustness of the estimates. The causal relationships of androsterone sulfate and glycine with ALM, hyodeoxycholate, glycine and 4-androsten-3beta, 17beta-diol disulfate 1 with the right HGS , and androsterone sulfate with WP were reliable and similar effect estimates were found for the weighted median, MR-Egger and MR-PRESSO method (Figure 4 and Supplementary file 1: Table S8). According to the results of the leave-one-out sensitivity analysis, hyodeoxycholate [β(95%) = 0.027(0.010, 0.044), P = 1.88×10^-3^] and glycine [β(95%) = -0.039(-0.060, -0.018), P = 2.35×10^-4^] increased was associated with decrease estimate of right HGS, and androsterone sulfate showed a significant negative associated with ALM[β(95%) = 0.057 (0.020,0.093), P = 2.19×10^-3^]. Therefore, the MR analysis was reliable, and no single SNPs changed the results substantially (Supplementary file 2: Figure S2-S5). |
|  | b) | Report results from other sensitivity analyses or additional analyses | 11-12 | Sensitivity analyses were conducted for the identified metabolites to evaluate the robustness of the estimates. The causal relationships of androsterone sulfate and glycine with ALM, hyodeoxycholate, glycine and 4-androsten-3beta, 17beta-diol disulfate 1 with the right HGS , and androsterone sulfate with WP were reliable and similar effect estimates were found for the weighted median, MR-Egger and MR-PRESSO method (Figure 4 and Supplementary file 1: Table S8). According to the results of the leave-one-out sensitivity analysis, hyodeoxycholate [β(95%) = 0.027(0.010, 0.044), P = 1.88×10^-3^] and glycine [β(95%) = -0.039(-0.060, -0.018), P = 2.35×10^-4^] increased was associated with decrease estimate of right HGS, and androsterone sulfate showed a significant negative associated with ALM[β(95%) = 0.057 (0.020,0.093), P = 2.19×10^-3^]. Therefore, the MR analysis was reliable, and no single SNPs changed the results substantially (Supplementary file 2: Figure S2-S5). |
|  | c) | Report any assessment of direction of causal relationship (e.g., bidirectional MR) | 10 | Fifty-four genetically predicted known metabolites associated with the components of sarcopenia were observed at the significance of P < 0.05 in the IVW analysis (Figure 2). As indicated by the results from the MR Steiger directionality test, the current estimates of causal direction were accurate (P < 0.001), and no SNP had shown pleiotropy (Supplementary file 1: Table S5). |
|  | d) | When relevant, report and compare with estimates from non-MR analyses | No Applicable |  |
|  | e) | Consider additional plots to visualize results (e.g., leave-one-out analyses) | 11-12 | According to the results of the leave-one-out sensitivity analysis, hyodeoxycholate [β(95%) = 0.027(0.010, 0.044), P = 1.88×10^-3^] and glycine [β(95%) = -0.039(-0.060, -0.018), P = 2.35×10^-4^] increased was associated with decrease estimate of right HGS, and androsterone sulfate showed a significant negative associated with ALM[β(95%) = 0.057 (0.020,0.093), P = 2.19×10^-3^]. Therefore, the MR analysis was reliable, and no single SNPs changed the results substantially (Supplementary file 2: Figure S2-S5). |
|  | **DISCUSSION** |  |  |  |
| 14 | **Key results** | Summarize key results with reference to study objectives | 12 | This study assessed the causal relationships between the blood metabolites and the sarcopenia-related traits through a MR study combining genomics and metabolomics. After the multiple-testing-adjusted Bonferroni correction, 3 known metabolites, which were pentadecanoate (15:0) on ALM, 3-dehydrocarnitine and isovalerylcarnitine on HGS were identified. Meanwhile, hyodeoxycholate and glycine were reliably negatively associated with the right HGS, and androsterone sulfate showed a reliable negative association with ALM in the sensitivity analysis. 13 metabolic pathways were identified to be causally associated with the components of sarcopenia. |
| 15 | **Limitations** | Discuss limitations of the study, taking into account the validity of the IV assumptions, other sources of potential bias, and imprecision. Discuss both direction and magnitude of any potential bias and any efforts to address them | 15-16 | This study has several limitations. Firstly, due to limited resources, no causal relationship has been identified between blood metabolites and sarcopenia diagnosed based on the cut-off values (42, 43). Because more phenotypic information cannot be used to study individuals, the results lacked the influence on body size and composition. Secondly, more IVs identified in GWAS might be needed to help accurately assess the genetic influence on metabolites. The third approximation of muscle mass in the UK Biobank used in the present study was measured using bio-impedance analysis (BIA), which may be less accurate than the values measured by other imaging detections, such as dual-energy x-ray absorptiometry (DXA), magnetic resonance imaging (MRI) and computed tomography (CT). In addition, demographic characteristics had not been considered in the present analyses, and the study was primarily limited to individuals of European ancestry, which limits the generalization of the findings. |
| 16 | **Interpretation** |  |  |  |
|  | a) | Meaning: Give a cautious overall interpretation of results in the context of their limitations and in comparison with other studies | 12 | This study assessed the causal relationships between the blood metabolites and the sarcopenia-related traits through a MR study combining genomics and metabolomics. |
|  | b) | Mechanism: Discuss underlying biological mechanisms that could drive a potential causal relationship between the investigated exposure and the outcome, and whether the gene-environment equivalence assumption is reasonable. Use causal language carefully, clarifying that IV estimates may provide causal effects only under certain assumptions | 13-15 | Pentadecanoate (15:0) was a dietary biomarkers for dairy-fat consumption (25), which also ADDIN EN.CITE (26) played a vital role in muscle metabolism and function (27). This is consistent to the findings in the current study...... |
|  | c) | Clinical relevance: Discuss whether the results have clinical or public policy relevance, and to what extent they inform effect sizes of possible interventions | 16 | These findings might have implications for the biological mechanisms of sarcopenia and targeted drug development for muscle health. |
| 17 | **Generalizability** | Discuss the generalizability of the study results (a) to other populations, (b) across other exposure periods/timings, and (c) across other levels of exposure | 15-16 | In addition, demographic characteristics had not been considered in the present analyses, and the study was primarily limited to individuals of European ancestry, which limits the generalization of the findings. |
|  | **OTHER INFORMATION** |  |  |  |
| 18 | **Funding** | Describe sources of funding and the role of funders in the present study and, if applicable, sources of funding for the databases and original study or studies on which the present study is based | 18 | Acknowledgments |
| 19 | **Data and data sharing** | Provide the data used to perform all analyses or report where and how the data can be accessed, and reference these sources in the article. Provide the statistical code needed to reproduce the results in the article, or report whether the code is publicly accessible and if so, where | 18 | Acknowledgments |
| 20 | **Conflicts of Interest** | All authors should declare all potential conflicts of interest | 19 | The authors declare that they have no conflict of interest. |

This checklist is copyrighted by the Equator Network under the Creative Commons Attribution 3.0 Unported (CC BY 3.0) license.

1. Skrivankova VW, Richmond RC, Woolf BAR, Yarmolinsky J, Davies NM, Swanson SA, et al. Strengthening the Reporting of Observational Studies in Epidemiology using Mendelian Randomization (STROBE-MR) Statement. JAMA. 2021;under review.

2. Skrivankova VW, Richmond RC, Woolf BAR, Davies NM, Swanson SA, VanderWeele TJ, et al. Strengthening the Reporting of Observational Studies in Epidemiology using Mendelian Randomisation (STROBE-MR): Explanation and Elaboration. BMJ. 2021;375:n2233.
