# Supplementary material for: The causal relationship of human blood metabolites with the components of Sarcopenia: a two-sample Mendelian randomization analysis
Source: BMC Geriatr. 2024 Apr 15;24:339. doi: 10.1186/s12877-024-04938-x (PMC11017669; doi:10.1186/s12877-024-04938-x)
Supplement: Supplementary file 2 — Supplementary Material 2 [file 12877_2024_4938_MOESM2_ESM.docx]

***Supplementary file 2***

The causal relationship of human blood metabolites with the components of sarcopenia: a two-sample Mendelian randomization analysis

Wenxi Peng ^1#^; Zhilin Xia ^1#^; Yaxuan Guo^1^; Linghong Li ^1^; Jianrong He ^2*^ ; Yi Su ^1*^

^1^ Key Laboratory of Molecular Epidemiology of Hunan Province, School of Medicine, Hunan Normal University, Changsha, Hunan, 410013, China

^2^ Division of Birth Cohort Study, Guangzhou Women and Children's Medical Center, Guangzhou Medical University, Guangzhou, Guangdong, 511436, China

*Corresponding authors:

Yi Su, E-mail: [alddle@hunnu.edu.cn](mailto:alddle@hunnu.edu.cn)

Key Laboratory of Molecular Epidemiology of Hunan Province, School of Medicine, Hunan Normal University, 371 Tongzipo Road, Yuelu District, Changsha 410000, China

Tel: 0731-88912466

Jianrong He, E-mail: [jianrong.he@bigcs.org](mailto:jianrong.he@bigcs.org)

Division of Birth Cohort Study, Guangzhou Women and Children's Medical Center, Guangzhou Medical University, Guangzhou, Guangdong, 511436, China

^#^ These authors have contributed equally to this work.

**Figure S1.** The causal associations (P < 0.05) of known metabolites with the components (right/left hand grip strength, walking pace and appendicular lean mass) of sarcopenia in the two-sample MR analysis.

**Figure S2.** Scatter plots of identified metabolites passed all sensitivity analyses on ALM. (A) MR effect size for hyodeoxycholate on the right HGS. (B) MR effect size for androsterone sulfate on ALM. (C) MR effect size for glycine on the right HGS.

**Figure S3.** Forest plots of LOO analysis for hyodeoxycholate on the right HGS.

**Figure S4.** Forest plots of LOO analysis for androsterone sulfate on ALM.

**Figure S5.** Forest plots of LOO analysis for glycine on the right HGS.

**Figure S1.** The causal associations (P < 0.05) of known metabolites with the components (right/left hand grip strength, walking pace and appendicular lean mass) of sarcopenia in the two-sample MR analysis.


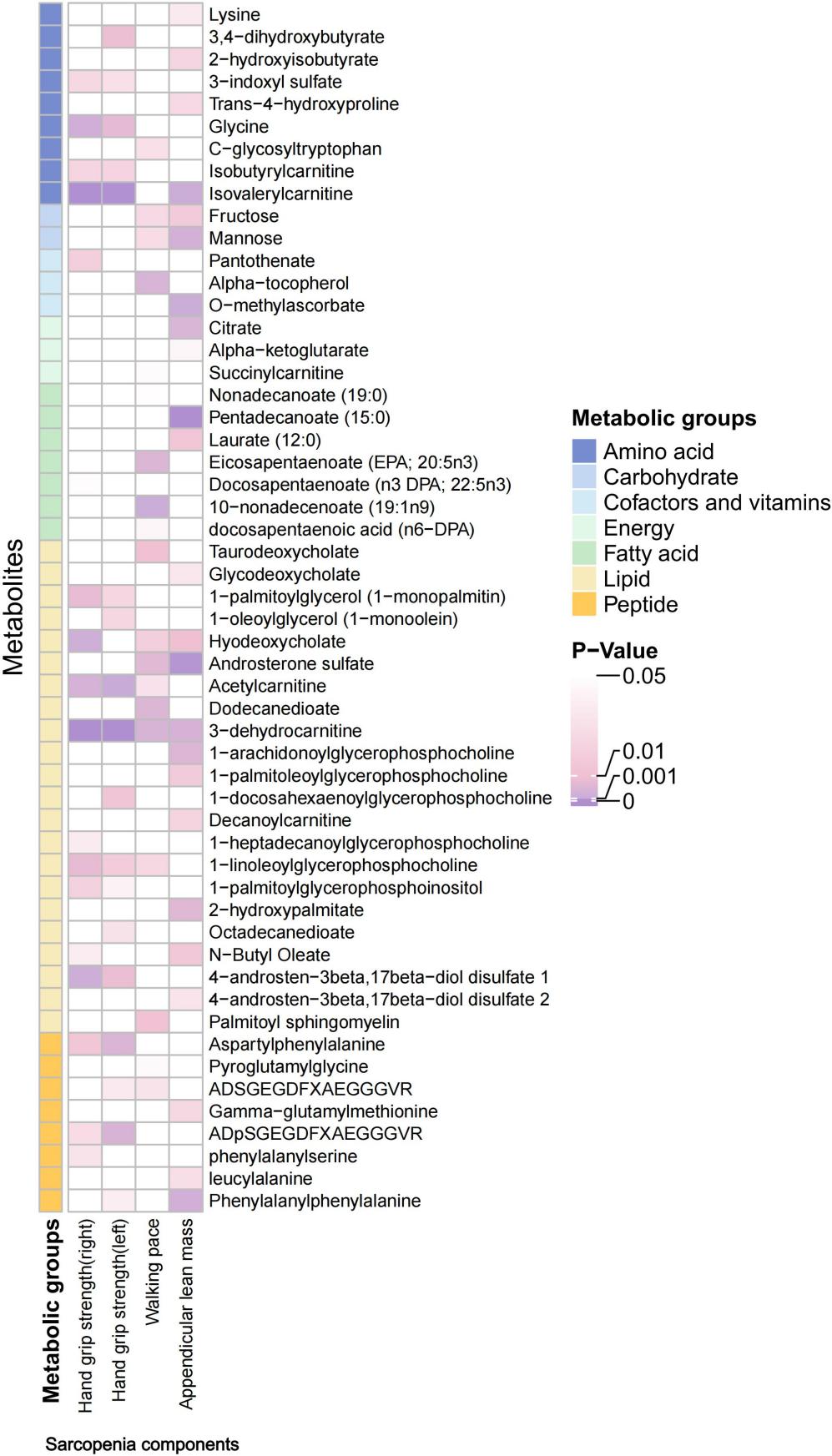


**Figure S2.** Scatter plots of identified metabolites passed all sensitivity analyses on ALM. (A) MR effect size for hyodeoxycholate on the right HGS. (B) MR effect size for androsterone sulfate on ALM. (C) MR effect size for glycine on the right HGS.


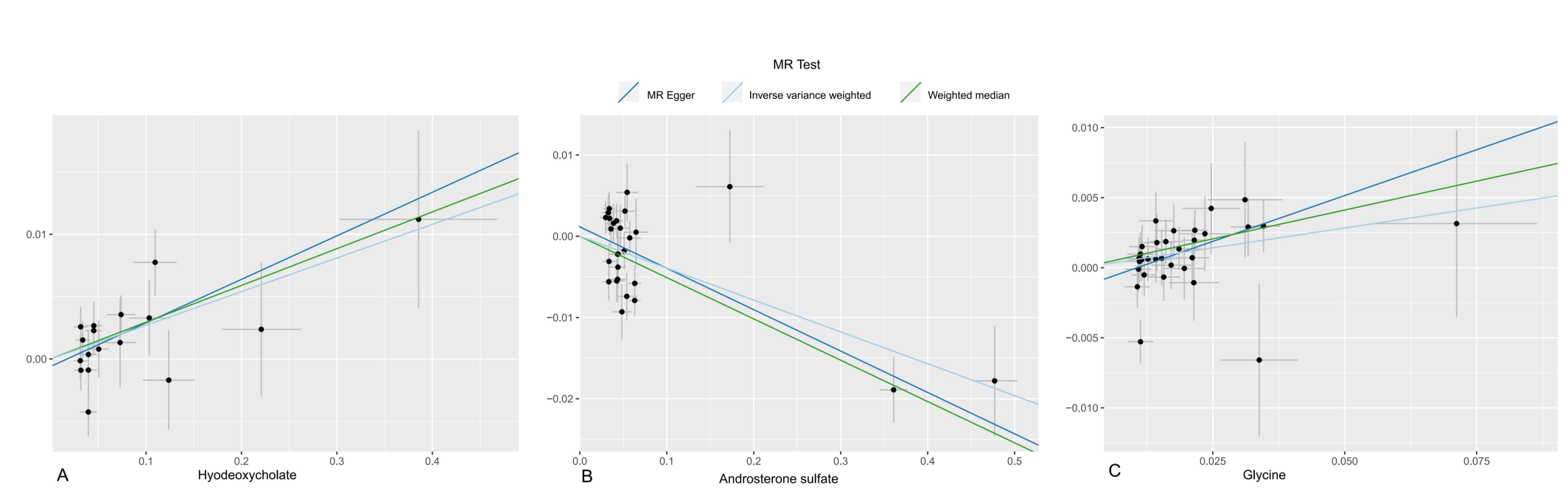


Notes:MR,Mendelian randomization; ALM, Appendicular lean mass; HGS,Hand grip strength.

**Figure S3.** Forest plots of LOO analysis for hyodeoxycholate on the right HGS.


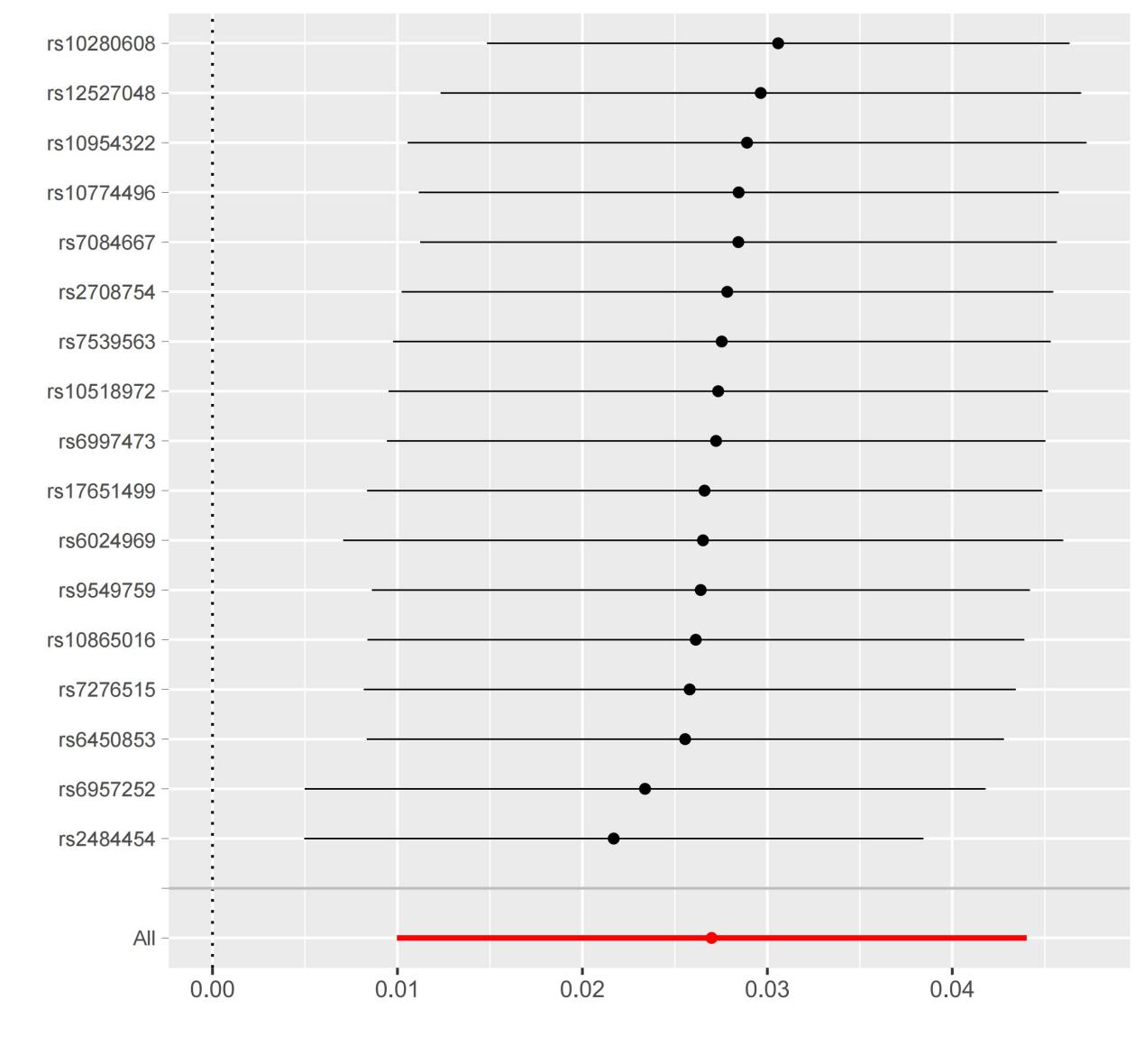


Notes:MR,Mendelian randomization; HGS,Hand grip strength; LOO analysis, Leave-one-out sensitivity analysis.

**Figure S4.** Forest plots of LOO analysis for androsterone sulfate on ALM.


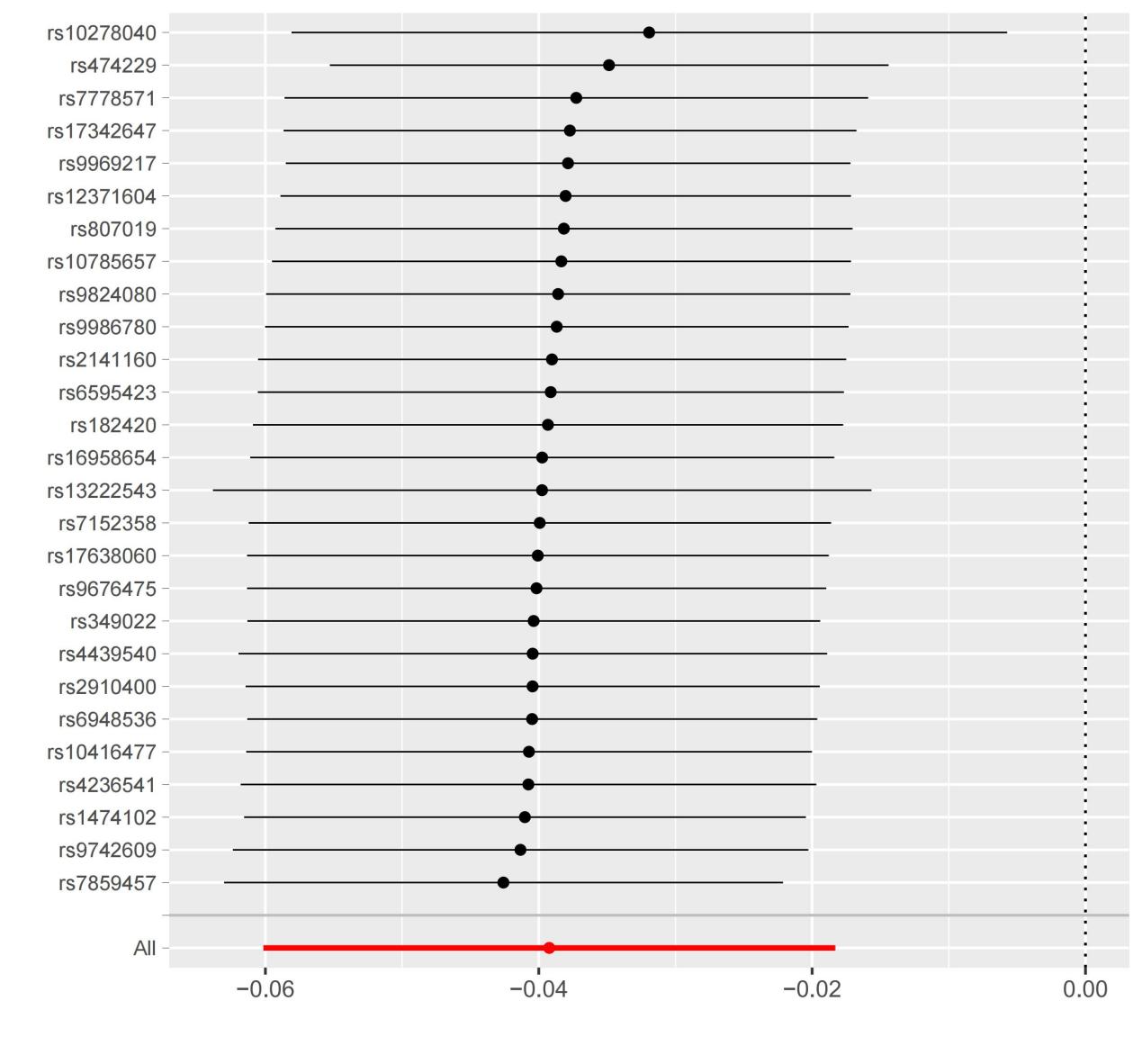


Notes:MR,Mendelian randomization; ALM, Appendicular lean mass; LOO analysis, Leave-one-out sensitivity analysis.

**Figure S5.** Forest plots of LOO analysis for glycine on the right HGS.


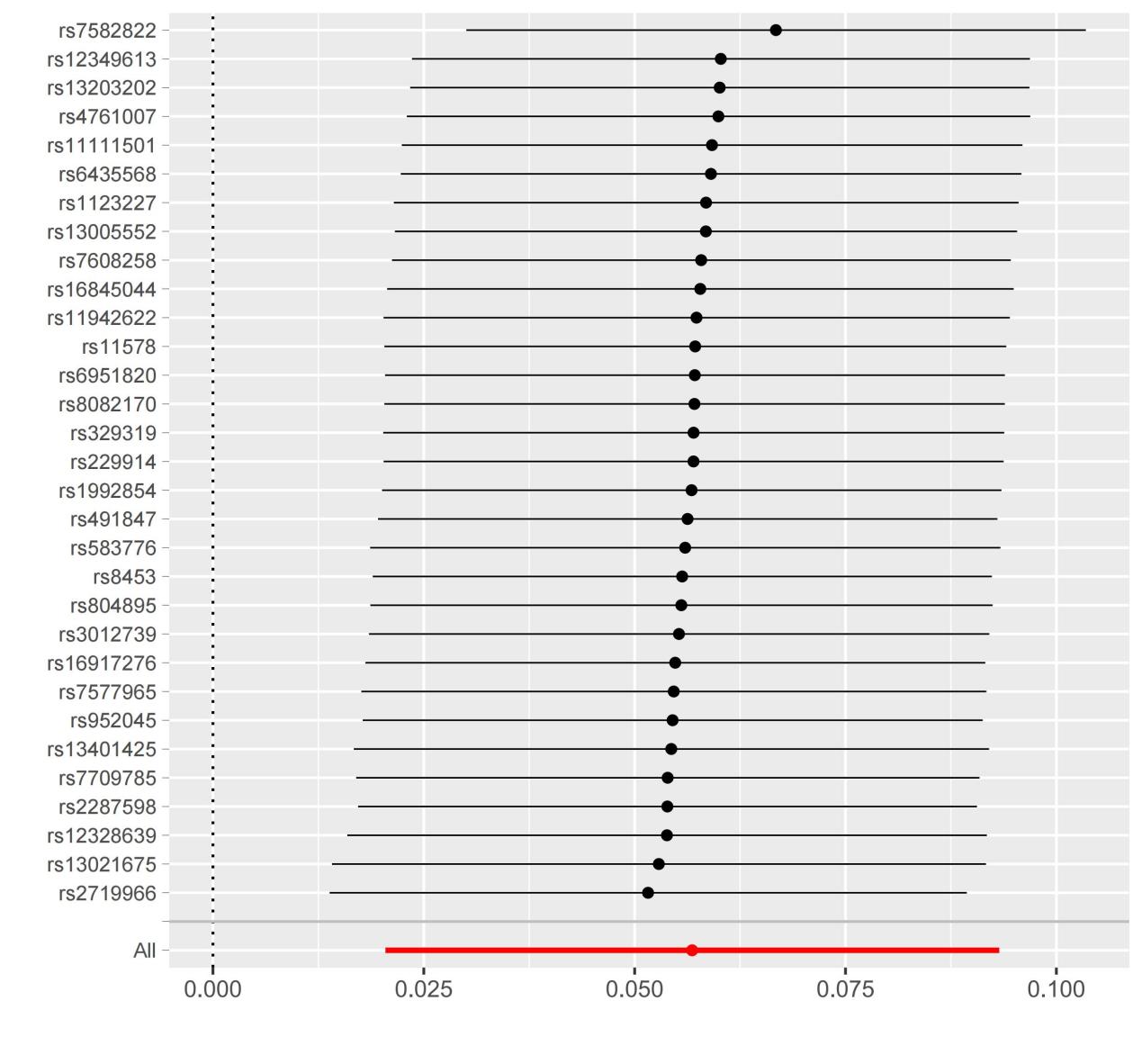


Notes:MR,Mendelian randomization; HGS,Hand grip strength; LOO analysis, Leave-one-out sensitivity analysis.
